# Comparative Volatile Metabolites and Aroma Profiles of *Geotrichum candidum* GC1 Cultivated in Three Media

**DOI:** 10.3390/metabo16100731

**Published:** 2026-09-29

**Authors:** Zhengrong Dou, Baorui Liu, Hongying Cai, Kun Meng, Yunsheng Han, Junpei Zhou, Weiwei Liu, Peilong Yang

**Affiliations:** 1 Laboratory of Feed Biotechnology of Ministry of Agriculture and Rural Affairs, Institute of Feed Research, Chinese Academy of Agricultural Sciences, Beijing 100081, China; zhengrongdou2020@163.com (Z.D.); liubr2001@163.com (B.L.); caihongying@caas.cn (H.C.); mengkun@caas.cn (K.M.); hanyunsheng@caas.cn (Y.H.); 2 Engineering Research Center for Valorization of Unique Bio-Resources in Yunnan, Ministry of Education, Yunnan Normal University, Kunming 650500, China; junpeizhou@ynnu.edu.cn; 3 School of Life Sciences, Yunnan Normal University, Kunming 650500, China; 4 Yunnan International Joint R&D Center for Sustainable Development and Utilization of Biological Resources, Yunnan Normal University, Kunming 650500, China

**Keywords:** *Geotrichum candidum*, corn steep liquor powder, volatile organic compounds, aroma-active compounds, E-nose, HS-SPME-GC-MS

## Abstract

Background/Objectives: Corn steep liquor Powder (CSLP) has previously been reported to promote fruity aroma production by *Geotrichum candidum*; however, the broader volatile profiles associated with CSLP-based cultivation relative to reference media remain insufficiently characterized. In this study, we compared the volatile metabolite and aroma-related profiles of *G. candidum* GC1 cultivated in a CSLP-based YMJ medium and two reference media, PDB and GAS. Methods: Using E-nose and HS-SPME-GC-MS, we compared the volatile profiles of *G. candidum* GC1 cultured in YMJ medium versus PDB and GAS media. Results: A total of 1414 volatile organic compounds (VOCs) belonging to 15 chemical classes were annotated. The YMJ group produced the highest VOC abundance, with significant enrichment of ketones, terpenoids, alcohols, pyrazines and phenols. Key compounds, such as 4-Nonenal (E) and 3(2H)-Furanone dihydro-2-methyl-, contributed fruity, sweet-bread-buttery-nutty notes, respectively, forming the complex floral-fruity-roasted aroma profile of *G. candidum* GC1 in YMJ medium. Volatile metabolomic analysis showed distinct medium-associated metabolic patterns, with the YMJ medium displaying a more diverse aroma-related volatile profile than the reference media under the conditions tested. Conclusions: The CSLP-based YMJ medium supported a distinct and relatively complex volatile profile in *G. candidum* GC1. These results support the potential use of CSLP-containing media in flavor-oriented fermentation design.

## 1. Introduction

Aroma is a decisive quality attribute of foods because volatile compounds strongly shape sensory perception, consumer preference, and product value, whereas conventional flavor production by chemical synthesis or natural extraction is often constrained by high cost, low yield, unstable raw material supply, and environmental burdens. Microbial fermentation and biotransformation therefore offer sustainable, cost-effective alternatives for tailoring volatile profiles and producing aroma-active compounds from renewable substrates, for example by overexpressing key shikimate pathway enzymes in yeast to enhance aromatic amino acid precursor supply and downstream aroma-active volatile formation [1,2]. The characteristic flavor of surface-ripened cheeses results from complex biochemical reactions, including lipolysis, proteolysis and amino acid catabolism, which generate a wide range of volatile organic compounds (VOCs) such as alcohols, esters, aldehydes, ketones and sulfur- or nitrogen-containing molecules [3]. Among the complex microbial consortia involved in cheese ripening, *Geotrichum candidum* is regarded as a key surface-ripening fungus that contributes to both the typical appearance and aroma of many soft and semi-soft cheeses [4]. Previous studies have shown that *G. candidum* can convert lipids and amino acids into aroma-active compounds with fresh cabbage-like and cheesy odors through multiple proteolysis- and lipolysis-related pathways, including the Ehrlich pathway, β-oxidation, alcohol acyltransferase-mediated esterification, and methionine catabolism, thereby playing an essential role in shaping the sensory profile of cheese [5,6,7,8].

The biosynthesis of volatile metabolites by filamentous fungi is strongly affected by environmental and nutritional conditions, particularly medium composition [9,10]. Carbon and nitrogen sources, mineral salts and growth factors can modulate fungal growth, enzyme activities and metabolic pathways, ultimately altering the quantity and balance of aroma-active compounds [11,12]. Corn steep liquor powder (CSLP), a major by-product of the corn wet-milling industry, is widely used as a low-cost nitrogen source and complex nutrient supplement in industrial fermentation. It is rich in peptides, free amino acids, organic acids, vitamins and minerals, and has been reported to enhance biomass formation and secondary metabolite production in various bacteria and fungi [13]. Corn steep liquor has been investigated as a nutrient source for aroma production by *G. candidum*. Notably, Pinotti et al. (2006) reported that medium components and amino acid supplementation, including corn steep liquor, influenced fruity aroma production by *G. candidum* [14]. However, the medium-dependent volatile metabolome of *G. candidum*, particularly the broad spectrum of VOCs and their potential aroma contributions under CSLP-based cultivation compared with chemically defined and conventional complex media, remains insufficiently characterized.

Accurate characterization of VOCs is crucial for understanding how culture conditions influence the flavor of fermented products. Gas chromatography–mass spectrometry (GC–MS) is widely recognized as a powerful analytical technique for identifying and quantifying volatile substances in complex matrices [15]. Nevertheless, GC–MS alone may not fully capture perceptual aroma differences and often requires laborious sample preparation and data interpretation [16]. In recent years, electronic nose (E-nose) systems based on metal oxide semiconductor sensors have been increasingly applied for the rapid evaluation of the overall volatile “fingerprint” of foods and beverages, including wines, beers and dairy products [16]. By combining E-nose measurements with multivariate statistical analysis, samples differing in raw materials, processing or microbial communities can be effectively discriminated according to their aroma profiles [17]. Integration of E-nose data and GC–MS results, for example those obtained by headspace solid-phase microextraction GC–MS (HS-SPME-GC–MS), provides comprehensive insights into both global odor patterns and specific aroma-active compounds [7]. However, there is still limited information on the application of such combined approaches to study the effects of culture media and CSLP on the volatile metabolites of *G. candidum* [4].

In our preliminary experiments, *G. candidum* GC1 cultivated in CSLP-containing YMJ medium produced a pronounced fruity aroma accompanied by mild roasted notes. This phenomenon suggested that the medium composition, especially CSLP as a complex nitrogen source, might play a crucial role in shaping the volatile profile of *G. candidum* GC1. To elucidate whether different media could modulate aroma formation, we selected commercial potato dextrose broth (PDB) and synthetic GAS medium, and compared them with CSLP-based YMJ medium. PDB is a commonly used nutrient-rich medium for filamentous fungi and provides suitable conditions for the growth and basal metabolism of *G. candidum*. In contrast, GAS medium uses glutamate as the sole nitrogen source and only differs from YMJ medium in nitrogen composition. The aims of this study were to compare the overall volatile characteristics of *G. candidum* GC1 grown in YMJ, PDB and GAS media and further combine E-nose detection with HS-SPME-GC–MS to identify key differentially accumulated metabolites (DAMs) and interpret their flavor characteristics. The results are expected to further enrich our understanding of the VOC profile produced by *G. candidum* GC1 cultured with CSLP, thereby providing a theoretical basis for the rational design of fermentation media and the regulation of flavor in fermented food.

## 2. Materials and Methods

### 2.1. G. candidum Media and Culture Conditions

*G. candidum* GC1 was isolated from traditional cheese and deposited in the China General Microbiological Culture Collection Center (CGMCC) under accession number CGMCC 42539. The strain was maintained on PDA (20 g/L glucose, 5 g/L yeast extract, and 15 g/L agar, Beijing Solarbio Science Technology Co., Ltd. (Beijing, China)) slants at 4 °C and preserved in 30% (*v*/*v*) glycerol solution at −80 °C for long-term storage. For activation, cultures were transferred to fresh PDA plates and incubated at 30 °C for 72 h [7].

Three fermentation media were used: PDB (5 g/L potato extract, 10 g/L peptone, 15 g/L glucose, 5 g/L NaCl, Beijing Solarbio Science Technology Co., Ltd. (Beijing, China)); synthetic GAS medium (25 g/L L-glutamate, 15 g/L glucose, 1.0 g/L KCl, 1.0 g/L MgSO_4_·7H_2_O, 2.5 g/L KH_2_PO_4,_ Beijing Solarbio Science Technology Co., Ltd. (Beijing, China)); and CSLP-based YMJ medium (25 g/L corn steep liquor powder (Shanghai Macklin Biochemical Co., Ltd. (Shanghai, China)), 15 g/L glucose, 1.0 g/L KCl, 1.0 g/L MgSO_4_·7H_2_O, 2.5 g/L KH_2_PO_4_). The pH of all the above media is natural. Mycelial plugs (10 mm in diameter) were inoculated into 100 mL potato dextrose broth (PDB) and incubated at 30 °C and 200 rpm for 24 h. Fresh mycelia were then collected by sterile filtration through a 400-mesh sieve and washed twice with sterile water. The mycelia were resuspended in 100 mL of sterile water, and 10% (*v*/*v*) of the suspension was inoculated into fresh medium for fermentation at 30 °C and 200 rpm for 48 h. The fermentation broths were centrifuged, and the supernatant was used for subsequent E-nose and GC-MS analyses. In addition, conPDB, conGAS, and conYMJ were the uninoculated PDB, GAS, and YMJ media, respectively, and served as the control groups for the E-nose analysis.

### 2.2. E-Nose Analysis

Volatile fingerprints were analyzed using a PEN3 electronic nose (AIRSENSE Analytics GmbH, Schwerin, Germany) equipped with 10 metal oxide sensors (Table 1). Sample preparation was adapted from Seesaard and Wongchoosuk with modifications [15]. Briefly, 15 mL of 48 h fermentation supernatant was transferred to a 50 mL centrifuge tube, sealed, and equilibrated at 25 °C for 2 h. Analysis parameters: sampling interval 1 s, pre-sampling 5 s, purge 60 s, sensor zeroing 5 s, injector flow 400 mL/min, detection time 120 s. Each sample, including the uninoculated control media, was analyzed in triplicate.

E-nose sensor responses were preprocessed using Microsoft Excel and WinMuster software. Radar charts were constructed using Microsoft Excel 2019. Multivariate analysis was performed with SIMCA 14.1 (Umetrics, Sartorius, Umeå, Sweden). Partial least squares discriminant analysis (PLS-DA) was applied to discriminate samples from different media. Model quality was assessed by R2X (explained variance), R^2^Y (dependent variable goodness-of-fit), Q^2^ (predictive ability), permutation testing (200 iterations), hierarchical clustering, and variable importance in projection (VIP) scores (VIP >1.0 was used to identify key variables), as described by Seesaard and Wongchoosuk [15].

### 2.3. GC-MS Analysis

Metabolomic analyses were performed in this study using Headspace Solid-Phase Microextraction-Gas Chromatography-Mass Spectrometry (HS-SPME-GC-MS) to analyze VOCs in three distinct fermentation samples. Frozen fermentation broth samples (−80 °C) were thawed on ice, and 0.2 mL aliquots were transferred to 20 mL headspace vials containing 0.2 g NaCl, sealed with TFE-silicone septa, and equilibrated at 60 °C for 5 min. Prior to injection, 20 µL of an internal standard solution (3-Hexanone-2,2,4,4-d4; catalog number D-8095-0.5g; C/D/N Isotopes Inc., Pointe-Claire, QC, Canada; purity 98%) at a concentration of 10 µg/mL was added to each sample. Volatiles were extracted using a 120 µm DVB/CWR/PDMS SPME Arrow (Model 8890; Agilent Technologies, Santa Clara, CA, USA) exposed to the headspace for 15 min at 60 °C, followed by thermal desorption at 250 °C for 5 min in the injection port of an Agilent GC coupled to a 7000D mass spectrometer (DB-5MS column: 30 m × 0.25 mm × 0.25 µm). The oven temperature was programmed from 40 °C (3.5 min), increasing to 100 °C at 10 °C/min, then to 180 °C at 7 °C/min, further to 280 °C at 25 °C/min, and finally held at 280 °C for 5 min. Mass spectra were acquired in EI mode (70 eV); ion source, quadrupole, and transfer line temperatures: 230 °C, 150 °C, and 280 °C, respectively. The mass spectrometer was operated in SIM mode to enable both qualitative and quantitative detection. Identification of volatile compounds was accomplished through matching retention times and mass spectra with a custom-built database constructed by Metware Biotechnology Inc., Wuhan, China (https://www.metware.cn/, accessed on 7 July 2026), which integrated data from literature references, commercial standards, and retention index information obtained from various biological samples [13,15,18]. For GC–MS analysis, each sample (YMJ, PDB, GAS) was analyzed in four replicates. The metabolomic data have been deposited in the China National Center for Bioinformation (CNCB) database under the accession number OMIX019285.

Multivariate statistical analysis was performed in R (www.r-project.org). Metabolite signal intensities were unit-variance scaled prior to unsupervised principal component analysis (PCA) using the prcomp function. Hierarchical cluster analysis (HCA) of samples and metabolites, together with Pearson correlation coefficients (PCC) between samples, were generated as heatmaps with dendrograms using the Complex Heatmap package (HCA was performed on unit-variance-scaled data). Differentially accumulated metabolites (DAMs) were screened using VIP > 1 from orthogonal partial least squares discriminant analysis (OPLS-DA) models, combined with |log_2_FC| ≥ 1.0 in pairwise comparisons. OPLS-DA models were built with the MetaboAnalystR package after log_2_ transformation and mean centering, and model robustness was validated by 200 permutation tests to rule out overfitting. Identified DAMs were annotated using the KEGG Compound database and mapped to KEGG pathways for enrichment analysis. The correlation analysis was performed using the Pearson correlation test by the Metware Cloud Platform (https://cloud.metware.cn, accessed on 7 July 2026).

### 2.4. Relative Quantification of VOCs

The relative content of VOCs was estimated using an internal-standard-based semi-quantification method as described by Huang and Yuan et al. [13,17]. The relative concentration of compound i (Xi, μg/mL) was calculated using the following equation:

Xi = (Vs × Cs/V) × (Ii/Is) × 10^−3^

where Xi is the content of compound i in the tested sample (μg/mL); Vs is the volume of internal standard (μL); Cs is the concentration of the internal standard (μg/mL); V is the volume of the tested sample (mL); Is is the peak area of the internal standard; Ii is the peak area of compound i in the sample; The factor 10^−3^ converts Vs from μL to mL to keep the unit system consistent.

### 2.5. Odor Activity Value (OAV) of VOCs

To assess the contribution of each VOC to the overall aroma profile, the relative OAV was calculated:

OAV = Ci/Ti

where Ci represents the relative concentration of compound i (estimated by the internal-standard method in Section 2.4), and Ti represents the odor threshold of compound i in water (same concentration unit as Ci). In general, compounds with OAV ≥ 1 are considered potential odorants that contribute directly to the sample aroma [13].

## 3. Results

### 3.1. E-Nose Analysis in GC1 After Fermentation

**Figure 1:**
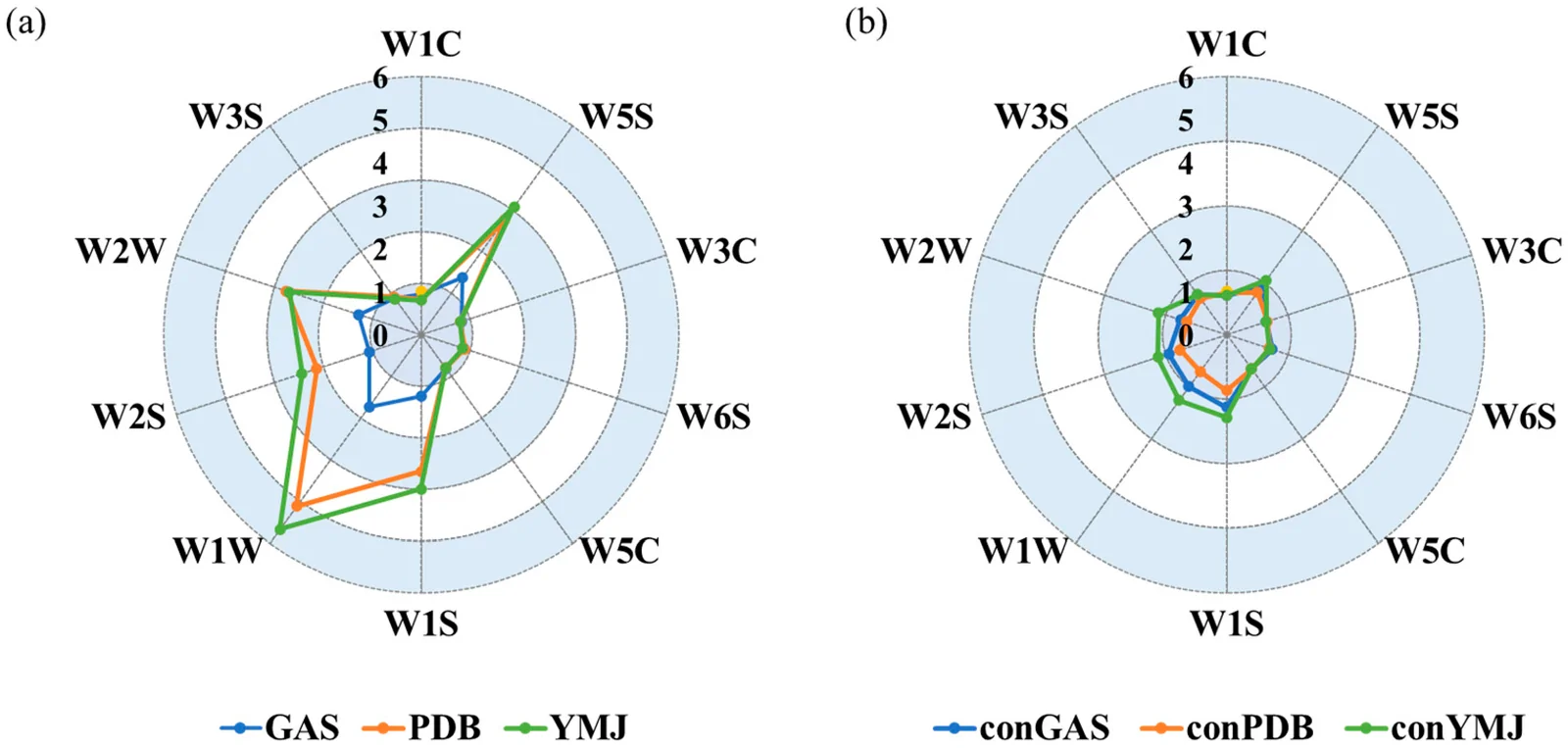
Radar chart showing electronic nose sensor responses to supernatant. (**a**) Supernatant of PDB, GAS, and YMJ groups; (**b**) Three types of blank control medium. The radar plots show the mean sensor responses from three independent replicates.

**Figure 2:**
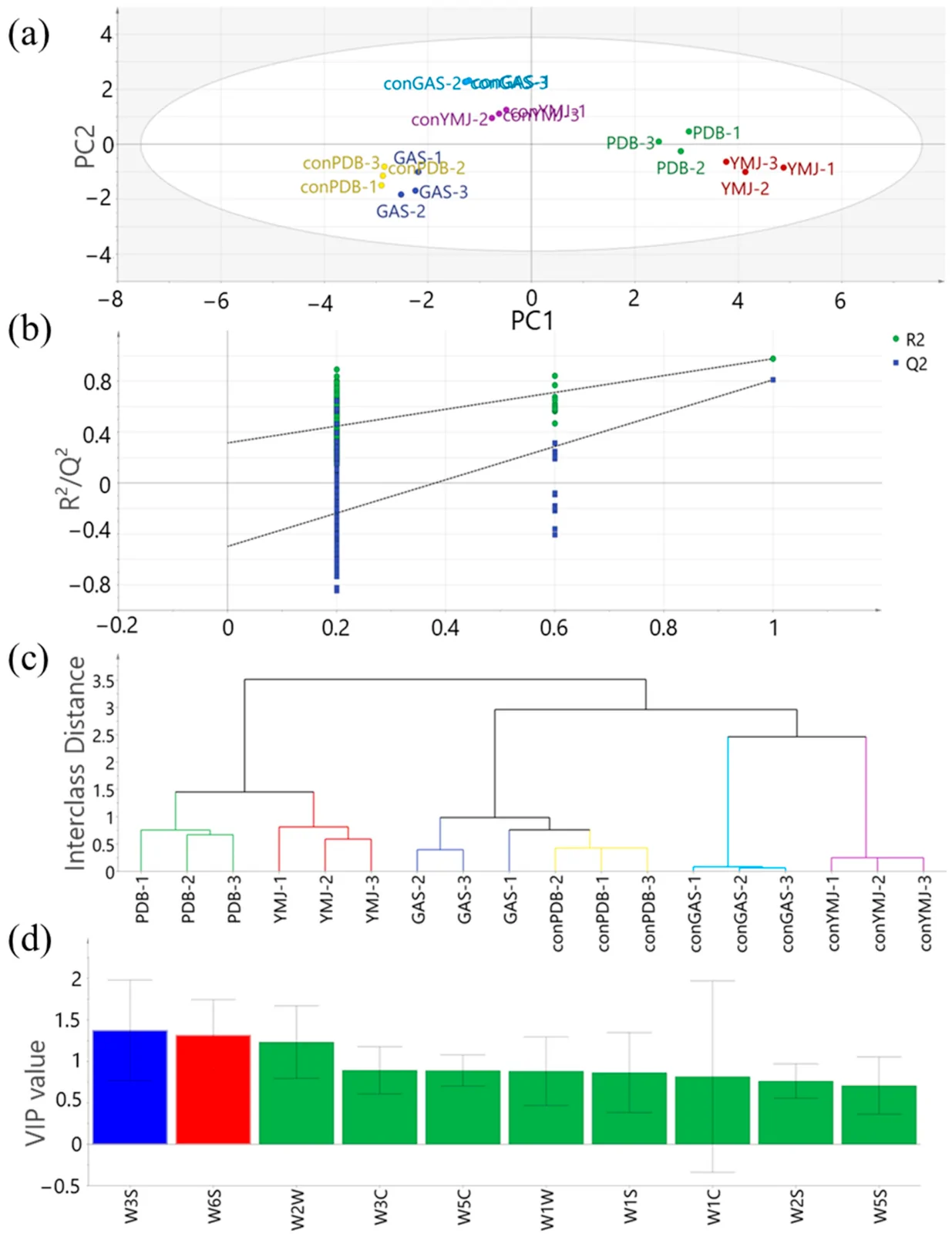
PLS-DA analysis of three kinds of fermentation broth and blank medium. (**a**) The score plot of PLS-DA showing clear group separation (R^2^X [1] = 73.8%, R^2^X [2] = 19.6%, Hotelling’s T^2^ = 95%). (**b**) Permutation validation model (R2 intercept = 0.278, Q2 intercept = −0.547). (**c**) Hierarchical clustering dendrogram. (**d**) VIP values. Sensors with VIP > 1 were identified as the key variables driving the discrimination among different groups.

After fermentation, pleasant odors were observed in the fermentation supernatants. The PDB, GAS, and YMJ groups exhibited fruity and wine-like, intense fruity, and mixed fruity–roasted aroma characteristics, respectively. To intuitively demonstrate the dominant volatile characteristics, a radar plot of E-nose sensor responses was generated, where the PDB, GAS, and YMJ groups showed relatively stronger responses to organic sulfides (W1W, W2W), nitrogen oxides (W5S), and short-chain alkanes (W1S) in the fermentation broths compared with the blank control group (Figure 1), indicating that *G. candidum* GC1 can utilize medium precursors to synthesize these VOCs. To further delineate aroma differences among media-specific fermentation broths of *G. candidum* GC1, PLS-DA and cluster analysis were performed on E -nose data. The PLS-DA clearly separated the three fermentation broths (R^2^X = 1.000, R^2^Y = 0.973, Q^2^ = 0.777), with the GAS showing more pronounced differences from the other two (Figure 2a). A 200-permutation test confirmed no overfitting, verifying model reliability (Figure 2b). Hierarchical Cluster analysis showed PDB and YMJ fermentation broths grouped into one branch while GAS formed a separate branch, further confirming significant differences in VOCs of the three fermentation broths (Figure 2c). Sensors with VIP > 1 (W3S, W6S, W2W) corresponded to differential substances including long-chain alkanes, hydrogen, and organic sulfides (Figure 2d). Although E-nose rapidly discriminates global aroma profiles, it lacks the capacity to identify individual aroma-active compounds. Figure 1 and Figure 2 showed no significant differences in the overall aroma profiles of the three control media. Nevertheless, compound-level differences may still exist but remain undetected by E-nose analysis. Since this study focused on the differences among PDB, GAS, and YMJ groups, GC–MS analysis was subsequently conducted only for these three groups to characterize the volatile compounds produced in each group.

### 3.2. GC-MS Analysis of VOCs in Three Media Fermented by G. candidum GC1

**Figure 3:**
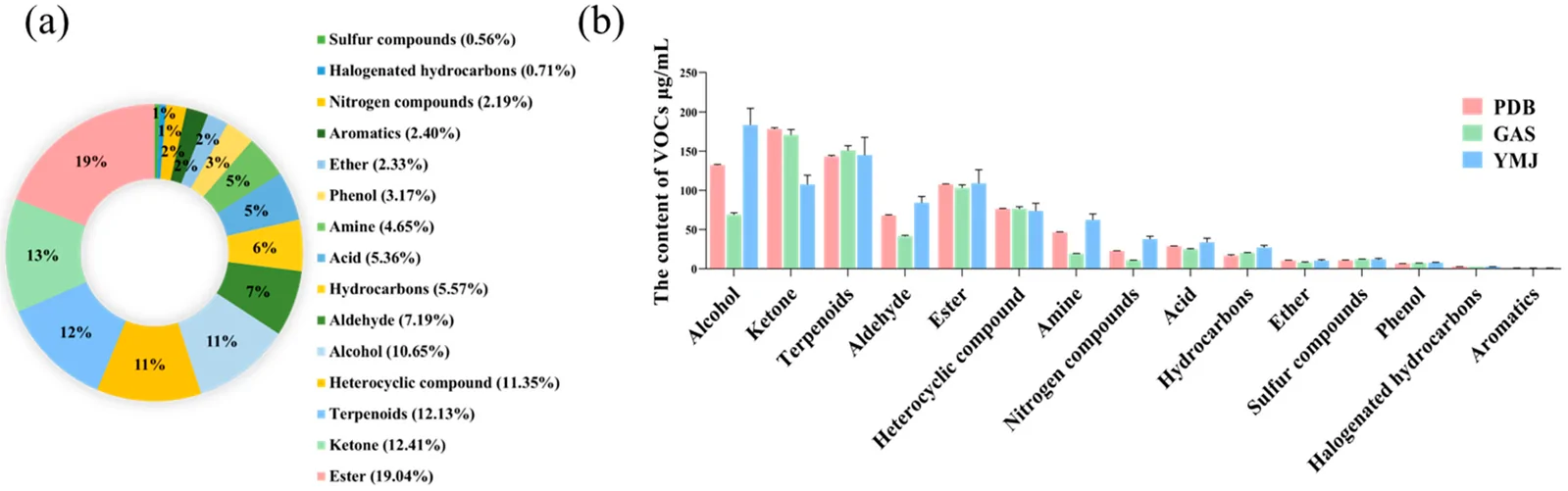
VOC analysis of three different supernatant groups by GC-MS. (**a**) Global distribution of detected VOCs across groups. (**b**) Comparison of VOC contents among YMJ, GAS, and PDB.

HS-SPME-GC-MS was employed to qualitatively analyze VOCs in three distinct fermentation samples. Using an internal standard semi-quantitative method, a total of 1414 VOCs were detected across three supernatant samples (Tables S1 and S2). All annotated VOCs were categorized into 15 classes (Figure 3a), including esters (270), ketones (176), terpenoids (172), heterocyclic compounds (161), alcohols (151), aldehydes (102), hydrocarbons (79), acids (76), amines (66), phenols (45), ethers (33), aromatic (34), nitrogen compounds (31), halogenated hydrocarbons (10) and sulfur compounds (8). As illustrated in Figure 3b, the total VOC profiles showed obvious differences among the three groups. For compound classes such as alcohols, aldehydes, amines and nitrogen compounds, the YMJ group showed higher contents, the PDB group displayed intermediate levels, and the GAS group had the lowest abundances. However, the total content of ketones in the YMJ group was lower than that in the PDB and GAS groups, whereas the total contents of terpenoids, esters, and heterocyclic compounds showed no obvious differences among the three groups.

### 3.3. Multivariate Statistical Analysis of DAMs

**Figure 4:**
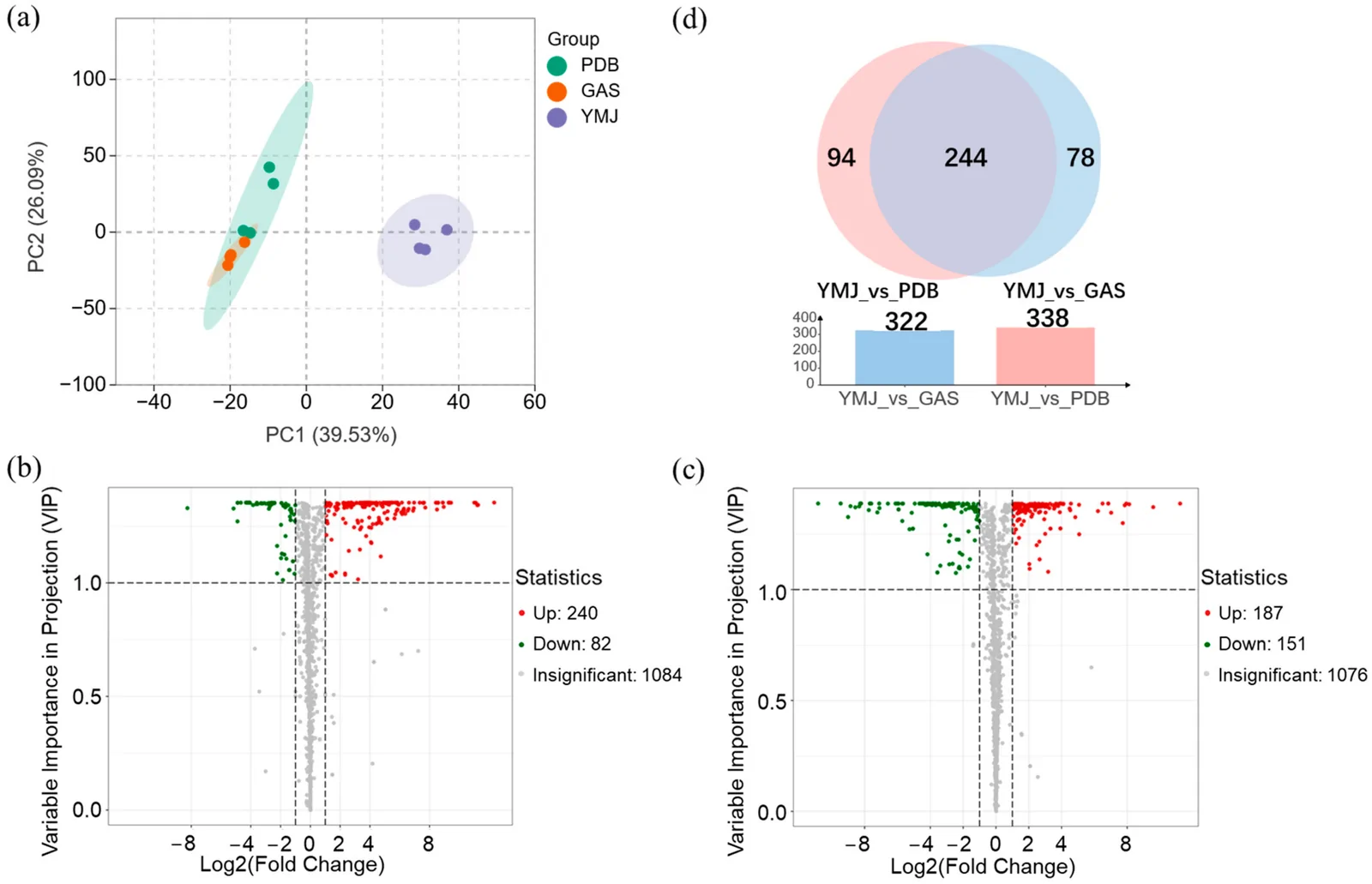
Volatile metabolite profiling of different fermentation broths fermented by *Geotrichum candidum* GC1. (**a**) PCA of samples from three fermentation broths. (**b**) Volcano plot of DAMs between YMJ and GAS. VIP > 1, |Log_2_FC| > 1.0. (**c**) Volcano plot of DAMs between YMJ and PDB. VIP > 1, |Log_2_FC| > 1.0. (**d**) Venn diagram showing the overlap of DAMs among the three fermentation broths. Each point in the volcano plot represents one metabolite. Green points indicate downregulated DAMs, red points indicate upregulated DAMs, and gray points represent detected metabolites that do not show statistically significant differences.

In sample quality control analysis, the total ion chromatograms (TICs) of different quality control (QC) samples exhibited high overlap during spectral detection. Over 75% of the compounds with coefficients of variation (CV) values below 0.3 demonstrated exceptional experimental stability and reliability (Figure S1). The VOCs in the three fermentation broths of *G. candidum* GC1 were significantly different. PCA revealed good biological reproducibility within the YMJ, GAS, and PDB groups and clear separation among them (Figure 4a), and this inter-group separation was further supported by hierarchical cluster analysis (Figure S2). The OPLS-DA model (Q^2^ = 0.999, *p* < 0.005, R^2^Y = 1.000, R^2^X = 0.912 of YMJ vs. GAS and R^2^X = 0.902 of YMJ vs. PDB) demonstrated excellent explanatory and predictive capabilities and could be further analyzed (Figure S3). A total of 322 DAMs were identified between YMJ and GAS (240 upregulated, 82 downregulated; Figure 4b); while 338 DAMs were detected between YMJ and PDB (187 upregulated, 151 downregulated; Figure 4c). A Venn diagram revealed 244 common DAMs between the YMJ vs. GAS and YMJ vs. PDB comparisons, with 94 metabolites specific to YMJ vs. PDB and 78 specific to YMJ vs. GAS (Figure 4d). Moreover, the diversity of DAMs between YMJ and PDB was higher than that between YMJ and GAS, further indicating that differences in medium composition strongly modulated the metabolic profile of *G. candidum* GC1 (Figure S4).

### 3.4. Potential Aroma-Active DAMs in the Three Fermentation Broths of G. candidum GC1

**Figure 5:**
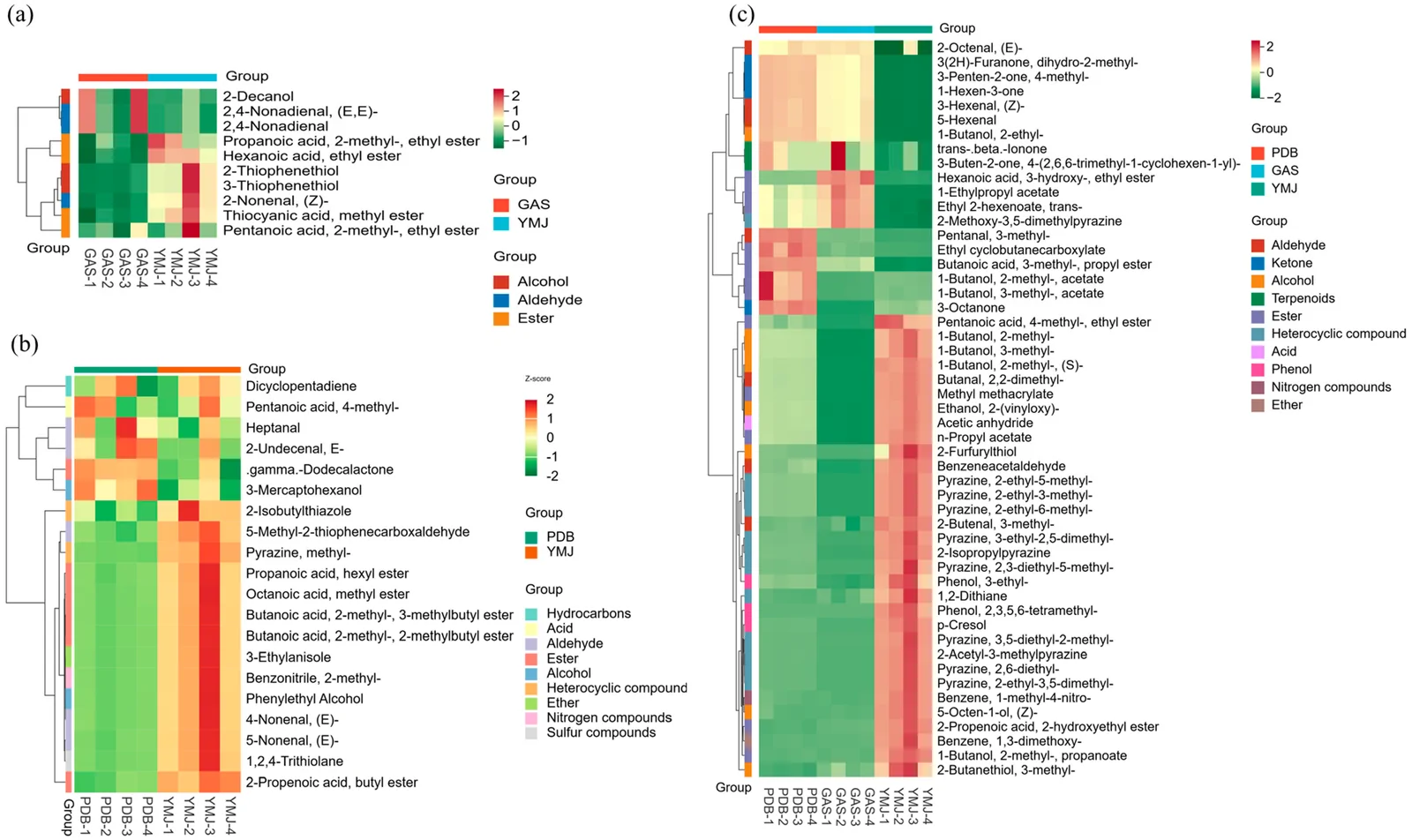
Heat map showing the OAVs of DAMs in different fermentation broths, OAV > 1. (**a**) DAMs unique to the YMJ vs. GAS comparison. (**b**) DAMs unique to the YMJ vs. PDB comparison. (**c**) Common DAMs between the YMJ vs. GAS and YMJ vs. PDB comparisons. The horizontal axis represents the sample names, whereas the vertical axis represents the information on the DAMs. The heatmap colors represent the normalized relative abundance values, with red indicating higher abundance and green indicating lower abundance. The clustering tree on the left represents the hierarchical clustering of the metabolites, and different colors indicate different metabolite categories. The metabolites included in the heatmap were selected based on VIP > 1.

OAV was used to quantify the contribution of individual aroma compounds to the overall aroma profile and identify potential flavor compounds in various foods [19]. Based on the analysis of OAVs, we characterized the flavor-forming DAMs in fermentation broths of *G. candidum* GC1 cultured in YMJ, PDB and GAS media, revealing that the YMJ group showed a prominent aroma advantage (Figure 5). The DAMs play a crucial role in the flavor development of *G. candidum* GC1 during fermentation across different media [14]. 10 compounds with OAV > 1 were identified among the 78 DAMs specific to YMJ vs. GAS (Figure 5a, Table S3). Among these, aldehydes and esters contributed most significantly to the flavor profile of the YMJ group. Specifically, 2,4-Nonadienal and 2,4-Nonadienal-(E,E) exhibited the highest OAVs, substantially imparting cucumber-like, green, fatty, and melon-fruity notes. Additionally, Propanoic acid 2-methyl- ethyl ester also displayed relatively high OAVs, contributing fresh fruity, sweet alcoholic, pineapple, and apple-skin-like aromas, accompanied by subtle sulfurous and ethereal notes. Furthermore, 2-Thiophenethiol and 3-Thiophenethiol enriched the YMJ group with cooked meat and garlic-like flavors. Meanwhile, Hexanoic acid-ethyl ester enriched the YMJ group with apple, pear, and fruity notes, and 2-Nonenal-(Z) contributed orris, fatty, waxy, and cucumber-like aromas.

20 compounds with OAV > 1 were identified among the 94 DAMs specific to YMJ vs. PDB (Figure 5b, Table S3). Aldehydes still acted as the most dominant aroma contributors. Among the DAMs shown in Figure 5b, 4-Nonenal-(E) showed an exceptionally high OAV in the YMJ group, representing a crucial flavor compound that primarily provided strong fruity notes. For alcohols, Phenylethyl alcohol with intense fruity, rosy, sweet, and apple-like odors displayed remarkably higher OAVs in YMJ, which greatly enriched the fruity aromatic layers. Additionally, several other classes of compounds also contributed to the unique flavor of YMJ. In terms of esters, Propanoic acid hexyl ester, Butanoic acid- 2-methyl- 2-methylbutyl ester, Butanoic acid 2-methyl- 3-methylbutyl ester and 3-Ethylanisole exhibited high OAVs in YMJ, contributing pear, green, sweet, berry, apple, and citrus-like fruity notes. 5-Methyl-2-thiophenecarboxaldehyde provided sweet, almond, cherry, and woody notes; 1,2,4-Trithiolane brought roasted beef and sulfury meaty notes; 2-Isobutylthiazole and Pyrazine methyl added green, vegetable, nutty, roasted, and cocoa-like notes.

Among the common 244 DAMs (Figure 5c), the ketone compound 3(2H)-Furanone dihydro-2-methyl was the primary aroma contributor in the three groups, followed by 3-Buten-2-one 4-(2,6,6-trimethyl-1-cyclohexen-1-yl) contributed floral, woody, sweet, fruity, berry, tropical, and beeswax characteristics. 1-Hexen-3-one imparted cooked, vegetable, and metallic aromas. 2-Methoxy-3,5-dimethylpyrazine and Pyrazine 2,3-diethyl-5-methyl contributed bread and mousy aromas, as well as a complex profile of musty, nut skin, earthy, roasted, hazelnut, toasted, potato, dusty, foliage, and vegetable notes. Meanwhile trans-.beta.-Ionone provided dry, powdery, floral, woody, and orris notes; Pyrazine 2-ethyl-3,5-dimethyl contributed burnt, almond, roasted, nutty, and coffee aromas; 1-Butanol 3-methyl was associated with whiskey, malty, and burnt characteristics; 2-Furfurylthiol stood out with sulfury, roasted, coffee, oily, fatty, burnt, and smoky notes. These compounds collectively define the sensory richness of the YMJ group, highlighting the diversity of flavor attributes driven by specific chemical classes. Furthermore, the other common DAMs with OAV > 1 were predominantly enriched in the YMJ group. These volatiles constituted a rich sensory matrix, encompassing sharp acidic, alcoholic, savory, green, floral, and roasted attributes driven by esters, ketones, alcohols, and aldehydes.

### 3.5. Correlation Between E-Nose and GC-MS

**Figure 6:**
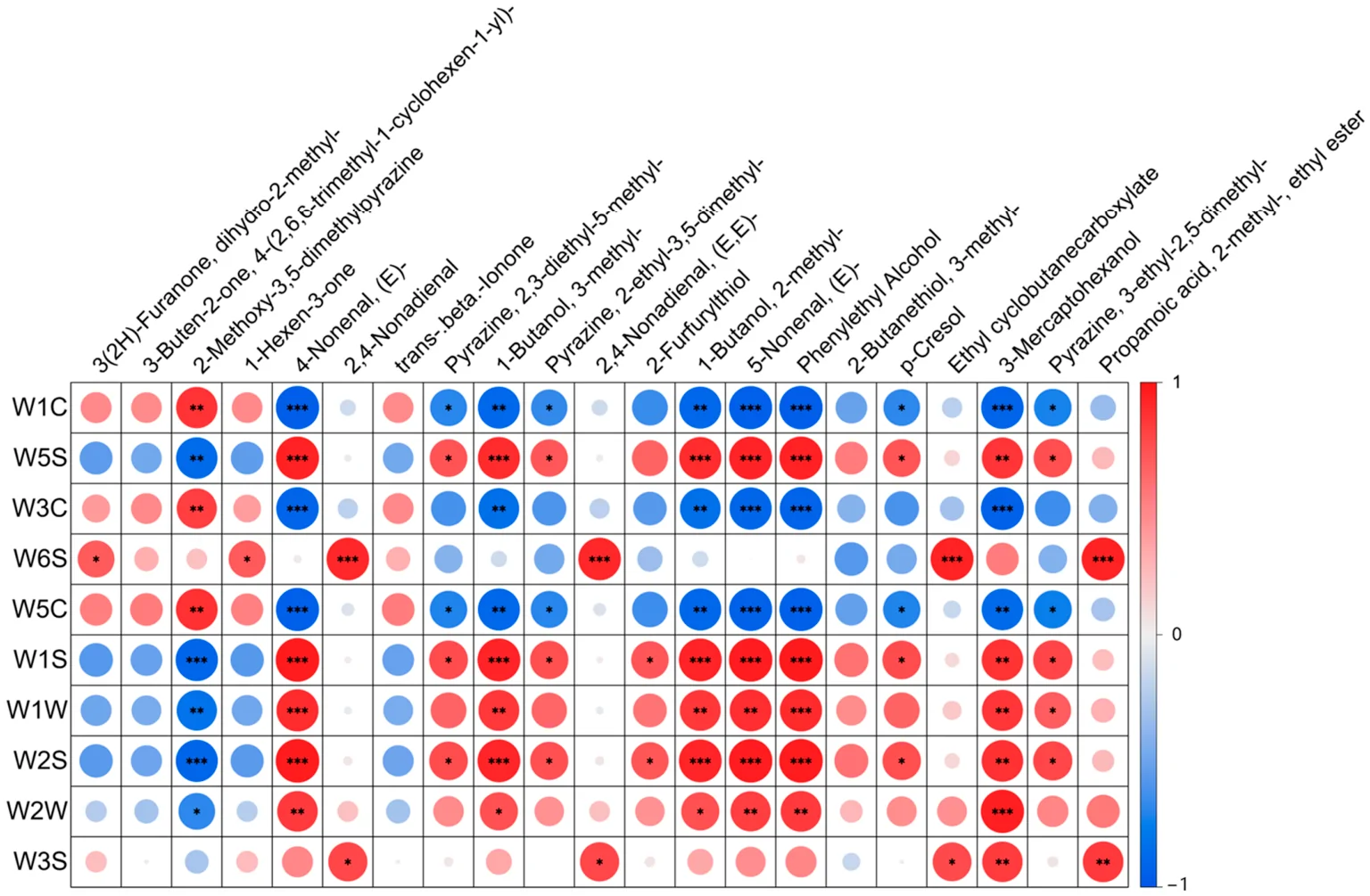
Correlations between E-nose sensors and the 21 DAMs detected by GC-MS. Significance levels: The color of the symbols within the cells reflects the strength of the correlation, with red indicating a positive correlation and blue indicating a negative correlation. When the symbols are ellipses, a larger ellipse indicates a greater absolute correlation coefficient, while a darker color also represents a greater absolute correlation coefficient. * *p* < 0.05, ** *p* < 0.01, *** *p* < 0.001.

To clarify the relationships between E-nose sensor responses and aroma-active VOCs, Pearson correlation analysis was conducted between the response values of the E-nose sensors and the VOCs annotated by GC–MS. Given that not all VOCs contribute significantly to the overall aroma, the top 21 compounds with the highest OAVs were selected as representative aroma-active VOCs for the correlation analysis. As shown in Figure 6, several key aroma-contributing compounds, 4-Nonenal-(E), 1-Butanol-3-methyl, 1-Butanol-2-methyl and Phenylethyl Alcohol, exhibited significant positive correlations with the sensors W5S (Broadrange), W1S (Broad methane), W1W (Sulphur organic), W2S (Broad alcohol) and W2W (Sulph chlor), and negative correlations with W1C, W3C and W5C. This finding is consistent with the radar plot results shown in Figure 1a, indicating the strong ability of these sensors to detect specific aroma-active compounds and their potential reliability in accurately characterizing the aroma profile. Moreover, compounds such as Ethyl cyclobutanecarboxylate, 3-Mercaptohexanol and Propanoic acid 2-methyl ethyl ester also exhibited significant positive correlations with W6S and W3S. W6S and W3S represent hydrogen and alkanes, respectively, and possess broad cross-sensitivity [20]. It is plausible that the reducing properties and alkyl chain structures of these three compounds activate the sensitivity of these sensors.

### 3.6. Distribution of DAMs Across Odor Descriptor Categories Based on OAV

**Figure 7:**
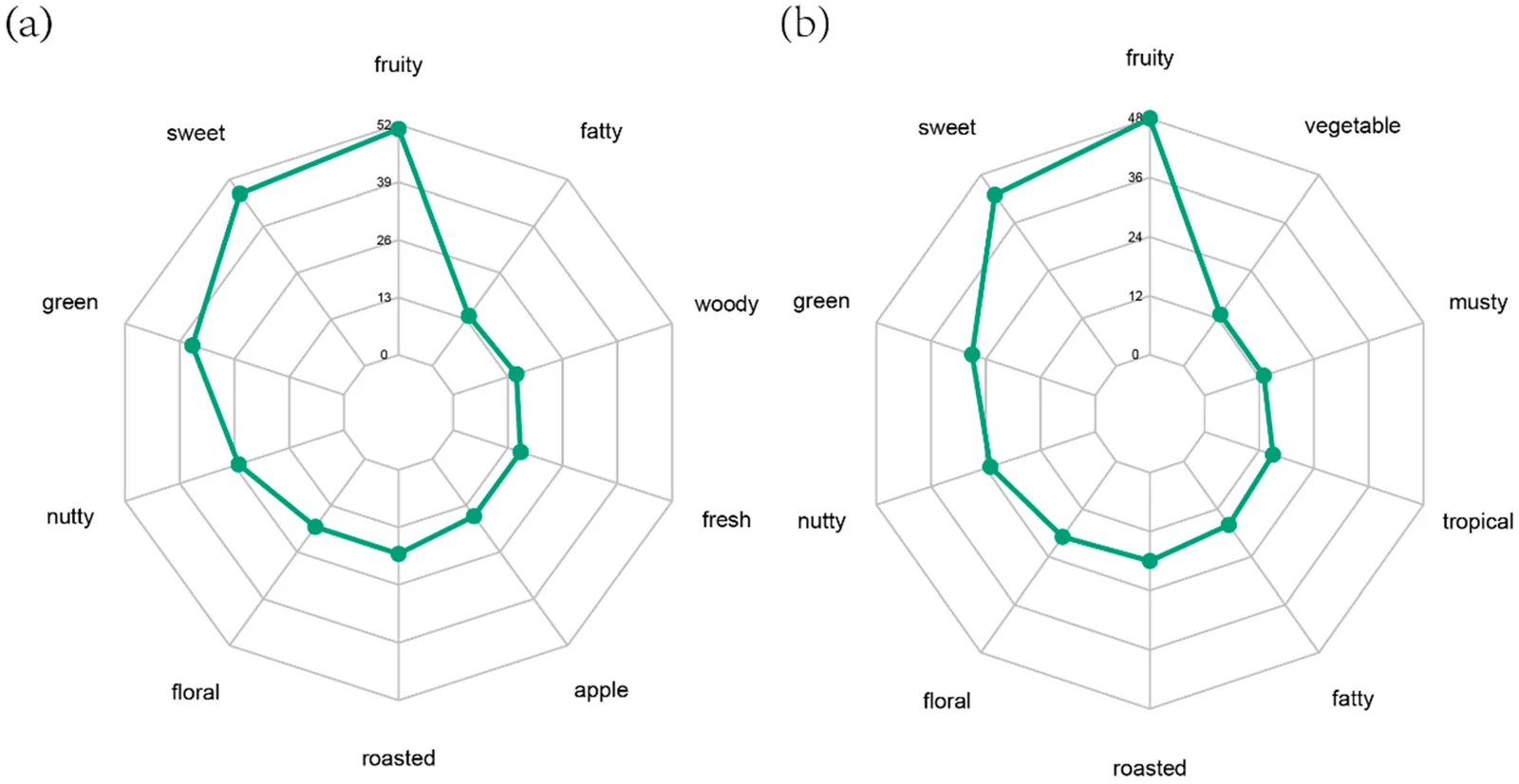
Radar charts for odor annotation of differential volatile metabolites. (**a**) YMJ vs. GAS comparison. (**b**) YMJ vs. PDB comparison. The axes represent the sensory odor descriptors, and the values marked by green dots indicate the number of differential metabolites annotated to each descriptor.

**Figure 8:**
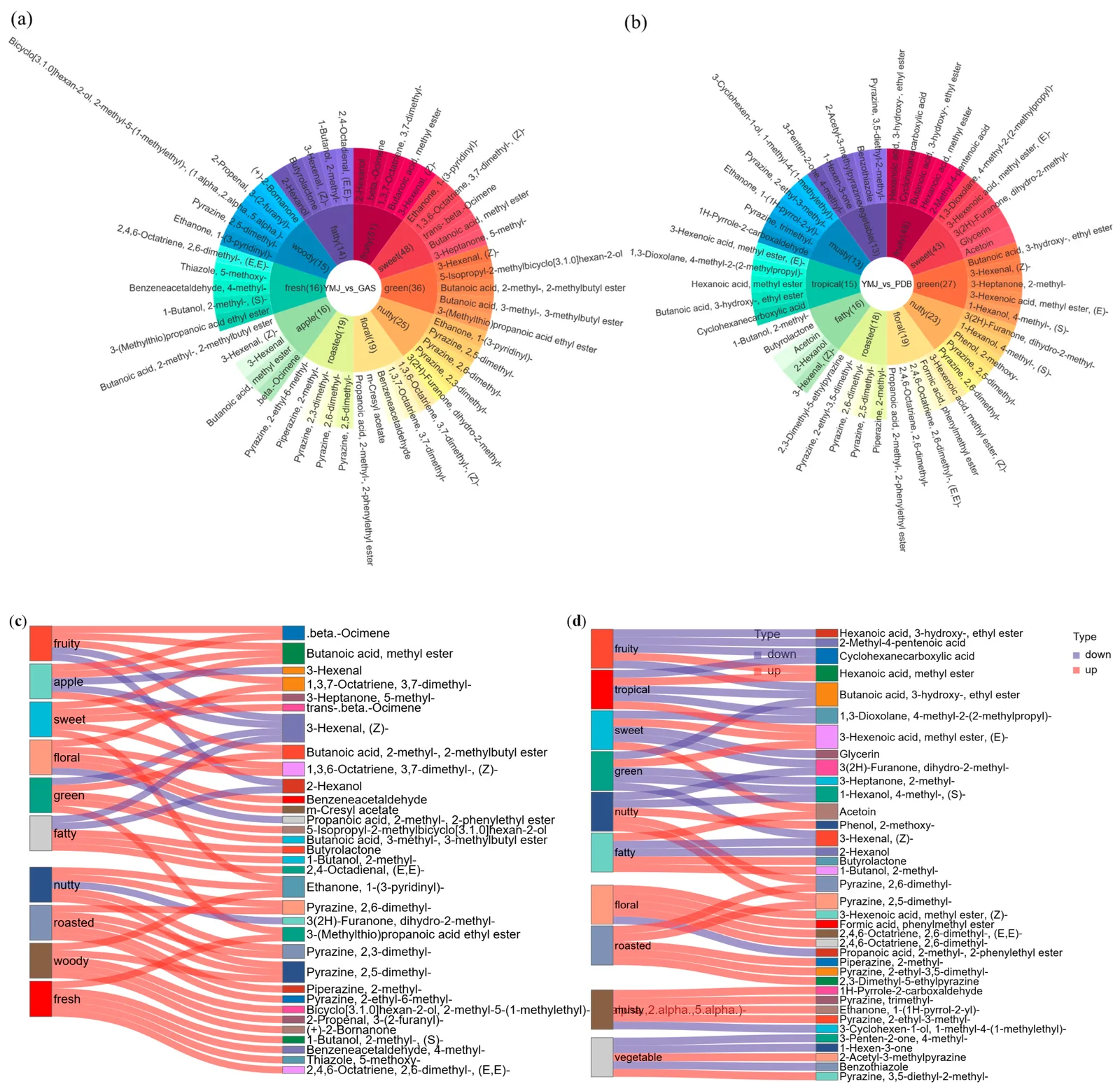
Flavor omics analysis of DAMs. (**a**) Flavor wheel of DAMs in YMJ vs. GAS comparison. (**b**) Flavor wheel of DAMs in YMJ vs. PDB comparison. (**c**) Sankey diagram between aroma and DAMs in group in YMJ vs. GAS. (**d**) Sankey diagram between aroma and DAMs in group in YMJ vs. PDB. VIP > 1.

To identify the odor-active basis underlying aroma differences, radar charts (Figure 7a,b) were used to provide an overview of the distribution of odor descriptors, whereas the sunburst plots (Figure 8a,b) and Sankey diagrams (Figure 8c,d) further linked these descriptors to specific DAMs. For the YMJ vs. GAS comparison, the radar chart showed that fruity and sweet were the odor descriptors associated with the largest numbers of compounds, followed by green, whereas nutty, floral, roasted, apple, fresh, woody, and fatty notes (Figure 7a). Consistently, the sunburst plot indicated that fruity (51), sweet (48), and green (36) were associated with the largest numbers of annotated DAMs (Figure 8a). The Sankey diagram further showed that multiple upregulated DAMs in YMJ group flowed into different odor dimensions (Figure 8c). For example, several metabolites associated with fruity/sweet characteristics, including beta-Ocimene, Butanoic acid methyl ester, 1,3,7-Octatriene 3,7-dimethyl, trans-.beta.-Ocimene, 3-Heptanone 5-methyl, 1,3,6-Octatriene 3,7-dimethyl (Z), Ethanone 1-(3-pyridinyl) were upregulated in YMJ. Meanwhile, Butanoic acid methyl ester also contributed to the apple attribute; 1,3,7-Octatriene 3,7-dimethyl, trans-.beta.-Ocimene, 1,3,6-Octatriene 3,7-dimethyl-(Z) were linked to floral notes; and Ethanone 1-(3-pyridinyl) was associated with nutty and woody notes. In addition, multiple pyrazines contributed to nutty/roasted notes, while Butanoic acid 2-methyl-2-methylbutyl ester and 3-(Methylthio)propanoic acid ethyl ester supported fresh/green nuances. Thus, these results indicate that YMJ exhibited a more complex, multi-faceted odor profile than GAS. For the YMJ vs. PDB comparison, the radar chart and sunburst plot similarly showed a profile dominated by fruity and sweet notes, followed by green, nutty, floral, roasted, fatty, tropical, musty, and vegetable attributes (Figure 7b and Figure 8b). In the Sankey diagram (Figure 8d), Hexanoic acid methyl ester, 3-Hexenoic acid methyl ester (E) and Acetoin were identified as upregulated DAMs contributing to fruity and sweet notes. Most of the other upregulated compounds, such as Phenol 2-methoxy, Pyrazine 2,6-diethyl, Pyrazine 2,5-dimethyl, 3-Hexenoic acid methyl ester-(Z), Formic acid phenylmethyl ester, 2,4,6-Octatriene-2,6-dimethyl-(E,E), 2,4,6-Octatriene-2,6-dimethyl and Piperazine-2-methyl were mainly linked to nutty, floral, and roasted attributes. Overall, YMJ showed a richer odor-active pattern than PDB. Taken together, these results suggest that YMJ was characterized by a more coordinated distribution of odor-active metabolites across multiple sensory dimensions, especially fruity, sweet, floral, nutty, roasted, and green notes.

### 3.7. KEGG Enrichment Analysis of DAMs

**Figure 9:**
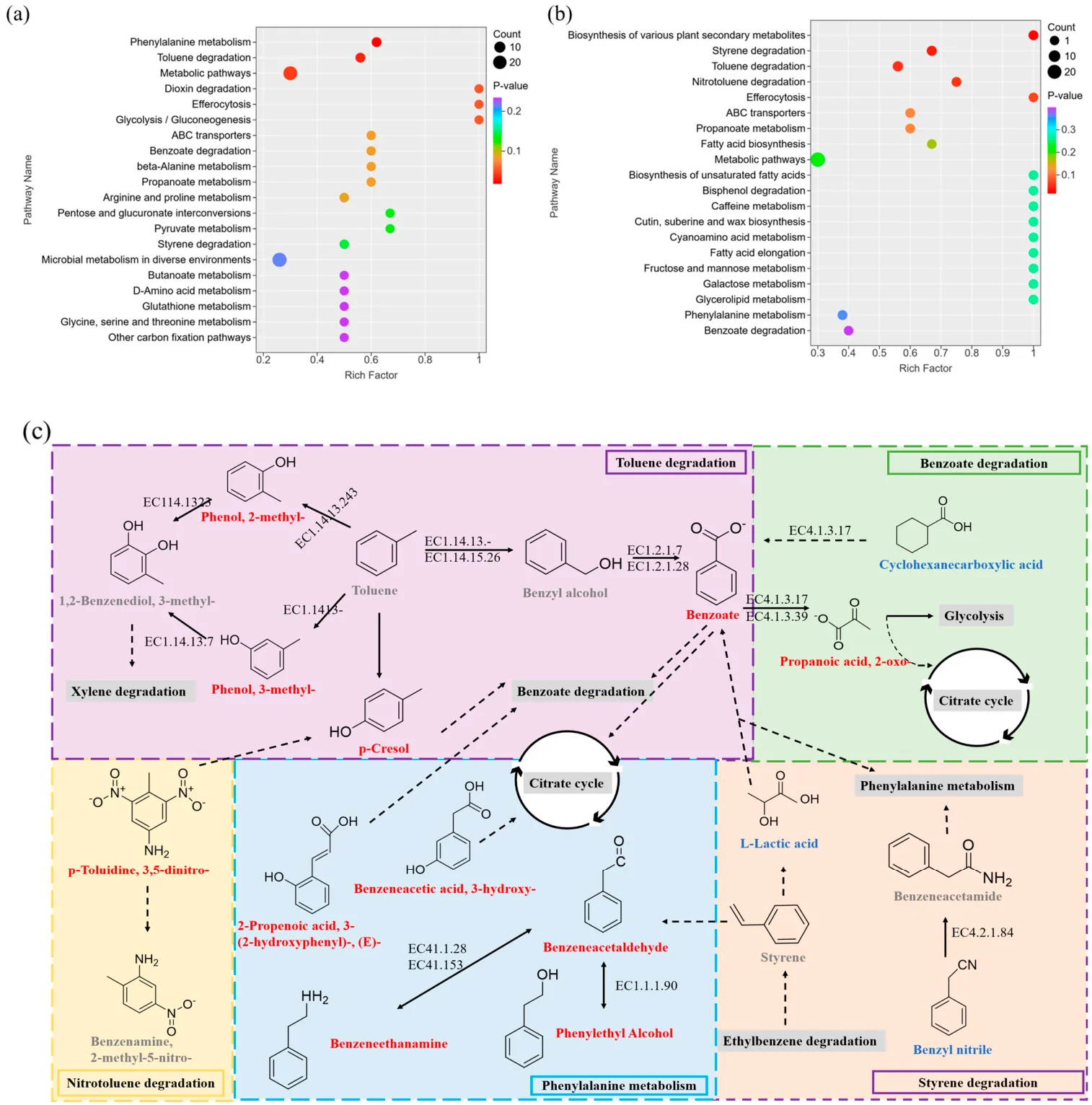
Metabolic pathway analysis of *G. candidum* GC1 cultured in the three media. (**a**,**b**) KEGG pathway enrichment bubble plots for YMJ vs. PDB and YMJ vs. GAS. (**c**) Integrated network of key aromatic metabolic pathways and their connections to central carbon metabolism. Red text indicates upregulated metabolites, and blue indicates downregulated metabolites and gray text indicates metabolites without significant change.

As shown in Figure 9a, for the YMJ vs. GAS comparison, phenylalanine metabolism, toluene degradation, and benzoate degradation were significantly enriched. Within the phenylalanine metabolism, 2-propenoic acid 3-(2-hydroxyphenyl)-(E), Benzeneacetic acid 3-hydroxy, phenylethyl alcohol, benzeneacetaldehyde, benzeneethanamine and Propanoic acid 2-oxo were significantly accumulated in the YMJ group (VIP > 1) (Figure 9c), contributing to the enrichment of this pathway (DA = 0.625, Figure S5). In addition, Propanoic acid 2-oxo was simultaneously annotated to the toluene degradation and benzoate degradation pathways, indicating crosstalk among aromatic metabolic networks [21]. Compared with GAS, most DAMs in YMJ showed an upward trend across the enriched pathways, suggesting a broader metabolic response of *G. candidum* GC1 in the nutrient-rich YMJ medium.

As shown in Figure 9b, for the YMJ vs. PDB comparison, biosynthesis of various plant secondary metabolites was annotated as the most significantly enriched pathway, followed by styrene degradation, toluene degradation, and nitrotoluene degradation. Overall, the DAMs mapped to these pathways exhibited both up- and down-regulated trends, with no dominant pattern (Figure S5). Several aromatic metabolites, including p-Toluidine-3,5-dinitro [22], Benzeneacetaldehyde [23] and Benzeneacetic acid 3-hydroxy-[24], were differentially accumulated between the two groups (Figure 9c), indicating broad alterations in aromatic metabolite profiles under the YMJ condition.

## 4. Discussion

*G. candidum* is widely recognized as an important flavor-producing microorganism in fermented foods because of its ability to generate diverse VOCs, including alcohols, ketones, esters, and sulfur-containing compounds [1,4]. In particular, sulfur flavor formation in *G. candidum* has been linked to amino acid catabolism and cystathionine γ-lyase activity, suggesting that nutrient composition can directly affect the biosynthesis of potent odorants [6]. In this study, the volatile profiles varied substantially among media, indicating that CSLP (YMJ medium), together with potato, peptone (PDB medium) and monosodium L-glutamate medium (GAS medium), jointly regulated aroma-related metabolic pathways. revealed stronger responses to organic sulfides (W1W, W2W), nitrogen oxides (W5S), and short-chain alkanes (W1S) (Figure 1). GC–MS annotated 1414 VOCs across the three media fermented by *G. candidum* GC1, with esters, ketones, terpenoids, and alcohols as the predominant classes (Figure 3a). Quantitative evaluation further demonstrated that the YMJ group yielded the highest total VOC concentration (Figure 3b). PCA and OPLS-DA clearly segregated the PDB, GAS, and YMJ groups, and the hierarchical clustering heatmap confirmed medium-specific volatile patterns. Concurrently, 338 DAMs were identified between YMJ vs. PDB groups, and 322 DAMs between YMJ vs. GAS groups (VIP > 1), revealing pronounced medium-specific chemical profiles, which is consistent with previous reports that nutrient composition modulated volatile diversity in *G. candidum* [7,14]. This is because CSLP of YMJ medium is a nutrient-rich by-product containing amino nitrogen, peptides, vitamins, and minerals, which may alter precursor availability, carbon/nitrogen balance, and microbial physiological status during fermentation [13]. Consequently, the observed differences in VOC profiles may reflect medium-associated effects and altered fungal metabolism; however, enhanced fungal biosynthesis cannot be distinguished from contributions originating from the media or medium-derived precursors under the current experimental design.

DAMs dominating the flavor contribution with OAV ≥ 1 were screened out by HS-SPME-GC-MS coupled with OPLS-DA modeling. In this study, ketones (3(2H)-Furanone dihydro-2-methyl and 1-Hexen-3-one) emerged as major contributors among the DAMs in YMJ medium fermented by *G. candidum* GC1, imparting prominent sweet, buttery and nutty notes. 3(2H)-Furanone dihydro-2-methyl, commonly known as coffee furanone, is a heterocyclic compound belonging to the furanone family. As a volatile natural flavour constituent of roasted coffee, it possesses insecticidal and antimicrobial activities [25,26]. Meanwhile, 1-hexen-3-one is recognized for its fresh creamy aroma, serving as a major flavor constituent in butter, milk, papaya, and corn [27,28,29,30]. Terpenoids (3-Buten-2-one 4-(2,6,6-trimethyl-1-cyclohexen-1-yl) and trans-.beta.-Ionone) contributed floral and fruity characteristics. Li et al. reported that 3-Buten-2-one 4-(2,6,6-trimethyl-1-cyclohexen-1-yl), one of the characteristic components in *Moringa oleifera*, contributes to the floral, woody, sweet, herbal and fruity aroma characteristics [31]. Heterocyclic compounds (2-Methoxy-3,5-dimethylpyrazine, Pyrazine 2,3-diethyl-5-methyl, Pyrazine 2-ethyl-3,5-dimethyl) enriched the profile with earthy, roasted, and coffee aromas. It has been reported that 2-methoxy-3,5-dimethylpyrazine was detected in *Butyriboletus roseoflavus* and *Citrus reticulata*, and was identified as a key contributor to the aroma formation [32,33]. Aldehydes (2,4-Nonadienal-(E,E), 2,4-Nonadienal, 4-Nonenal-(E)) further intensified fatty, melon and chicken-like impressions, and 4-Nonenal-(E) serves as a critical node in the lipid oxidation pathway, thereby mitigating the formation of fishy off-odors [34]. Alcohols (Phenylethyl alcohol, 1-Butanol 3-methyl-, 2-Furfurylthiol) added fruity, rose, whiskey and malty dimensions. Ester (Propanoic acid 2-methyl- ethyl ester) contributed fruity, sweet and ethereal notes, and it is frequently employed as an edible flavoring agent in the formulation of fruit-flavored products [35]. Collectively, these volatiles synergistically generated the distinctive complex fruity-roasted aroma of YMJ fermentation broths of *G. candidum* GC1, spanning sharp acidic, alcoholic, savory/meaty, green/earthy, floral/fruity and roasted/nutty layers that markedly surpassed the simpler profiles observed in PDB and GAS media (Figure 5, Table S3). This medium-driven aroma diversification aligns closely with previous reports. For instance, Mdaini et al. demonstrated that the composition of the culture medium significantly influences the production of volatile compounds by *G. candidum*, leading to distinct fruity and other aroma profiles [7]. Additionally, Pinotti et al. reported that the supplementation of corn steep liquor selectively enhances the biosynthesis of esters and alcohols, which results in the intensification of fruity notes [14]. In cheese systems, Gente et al. confirmed that cystathionine γ-lyase-mediated sulfur metabolism produces the meaty, roasted and garlic-like volatiles enriched here [6].

KEGG enrichment analysis indicated that the YMJ medium significantly affected aromatic metabolism-related processes in *G. candidum* GC1. Significant enrichment was observed in pathways involved in secondary metabolite biosynthesis, phenylalanine metabolism, and several pathways related to aromatic compound transformation, suggesting that the flavor differences among media were driven by coordinated changes in multiple interconnected metabolic routes rather than a single pathway [36,37]. The accumulation of phenylethyl alcohol, benzeneacetaldehyde, benzeneethanamine, and Propanoic acid 2-oxo in YMJ suggests enhanced aromatic amino acid conversion, especially within phenylalanine metabolism. In fungi and yeasts, these transformations are often associated with enzymes such as aminotransferases, decarboxylases, alcohol dehydrogenases, and aldehyde dehydrogenases, which participate in the conversion of aromatic amino acids into flavor-active aldehydes and alcohols [38]. Although enzyme activities were not measured in this study, the metabolite profile implies that YMJ may favor this type of flavor-associated conversion process. At the same time, several KEGG pathway categories related to aromatic compound metabolism were significantly enriched among the detected metabolites, including toluene degradation, styrene degradation, and benzoate degradation. Tese pathways are primarily functionally characterized in bacteria, where they degradation of aromatic pollutants [39,40]. However, in the present study, the enrichment pathway more likely reflect shared metabolites or reactions associated with broad aromatic-compound transformation. This is supported by the overlap of several metabolites among the enriched pathways and by the accumulation of Propanoic acid 2-oxo, implying a link between aromatic metabolism and glycolysis/TCA-related metabolism [41]. Overall, YMJ appears to promote flavor-associated metabolism in *G. candidum* GC1 by strengthening both phenylalanine-derived aromatic metabolite formation and aromatic compound degradation, while also facilitating their integration with central carbon metabolism. Further transcriptomic and enzymatic analyses will be needed to verify the specific genes and enzymes involved.

The correlation between E-nose sensor responses and GC–MS-derived OAV values indicated a reasonable consistency. Aldehydes, alcohols, and chlorine-containing compounds were mainly associated with sensors sensitive to broad-range, methane, alcohols, and sulphur-chlorine compounds, whereas esters and other alcohols exhibited cross-sensitivity with hydrogen and alkane sensors in a broad-response manner. These results suggest that the E-nose was effective in capturing medium-dependent differences in the overall volatile pattern of *G. candidum* GC1, which is consistent with its established role in rapid aroma discrimination and food quality evaluation [23,24]. Nevertheless, due to the cross-sensitivity of E-nose sensors, these associations should be regarded as indicative of global aroma pattern recognition rather than direct compound-specific detection [14]. Thus, E-nose and GC–MS were complementary in evaluating the aroma characteristics of different culture media.

## 5. Conclusions

In conclusion, the combined application of E-nose analysis, HS-SPME-GC-MS, and OAV evaluation demonstrated that CSLP-based YMJ medium supplementation and culture medium composition significantly reshaped the volatile secondary metabolism of *G. candidum* GC1. Changes in the medium formulation not only altered the overall odor fingerprint of the fermented samples but also influenced the accumulation of odor-active VOCs that are directly associated with flavor perception. These findings indicate that precursor availability and nutritional balance are key determinants of aroma formation in *G. candidum* GC1. More importantly, the study suggests that CSLP can act as both a low-cost nutrient source and an effective regulator of flavor development. Overall, this work provides new insight into the relationship between medium composition and volatile metabolite synthesis in *G. candidum*, and lays a theoretical foundation for the rational design of fermentation media to regulate flavor profiles of fermented food products.

## Figures and Tables

**Table 1:** Electronic-nose sensors and their sensitivity profiles.

Sensor	Performance Category	Specific Sensitivity Description
W1C	Aromatic	Aromatic components, benzene series
W5S	Broadrange	High sensitivity; sensitive to nitrogen oxides
W3C	Aromatic	Ammonia water; sensitive to aromatic components
W6S	Hydrogen	Mainly selective to hydrogen
W5C	Arom-aliph	Alkane and aromatic components
W1S	Broad-methane	Sensitive to short-chain alkanes (e.g., methane)
W1W	Sulphur-organic	Sensitive to organic sulfides
W2S	Broad-alcohol	Sensitive to alcohols, ethers, aldehydes and ketones
W2W	Sulph-chlor	Aromatic components; sensitive to organic sulfides
W3S	Methane-aliph	Sensitive to alkanes (long-chain alkanes in particular)

Note: The information listed in the table represents the manufacturer-reported sensitivity ranges.

## Data Availability

The raw metabolome sequencing data generated in this study have been deposited in the CNCB database (accession number: OMIX019285). All additional data supporting the validation of the conclusions of this paper are available in the main text and Supplementary Material.

## Supplementary Materials

The following supporting information can be downloaded at: https://www.mdpi.com/article/10.3390/metabo16100731/s1, Figure S1: Demonstrates the excellent stability and reproducibility of quality control (QC) sample analysis, Figure S2: Hierarchical cluster analysis of DAMs across all samples, Figure S3: Validation of the OPLS-DA model, Figure S4: Trend clustering of DAMs in the three fermentation broths of *G. candidum* GC1, Figure S5: Differential abundance score plots for the overall analysis of metabolic pathway changes, Table S1: All volatile metabolites annotated by GC-MS, Table S2: Volatile Organic Compounds (VOCs) Detected in PDB, GAS and YMJ Fermentation Samples, and Table S3: Summary of Differentially Accumulated Metabolites (DAMs) between YMJ, GAS and PDB Groups.
