# Supplementary material for: Comparative Volatile Metabolites and Aroma Profiles of Geotrichum candidum GC1 Cultivated in Three Media
Source: Metabolites. 2026 Sep 29;16(10):731. doi: 10.3390/metabo16100731 (PMC13706846; doi:10.3390/metabo16100731)
Supplement: Supplementary file 1 [file metabolites-16-00731-s001.zip › Supplementary File S2.pdf]

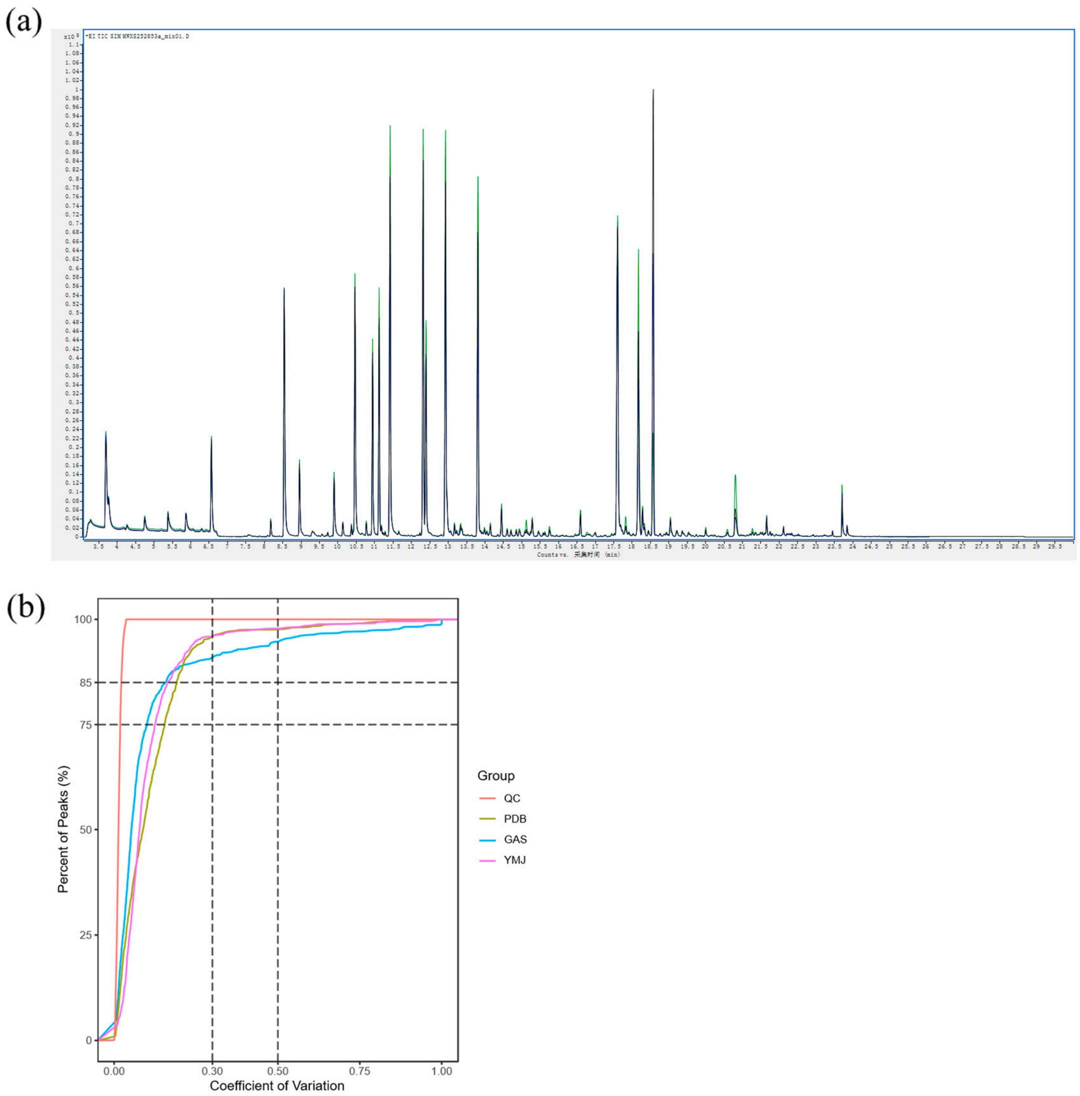

Figure S1. The excellent stability and reproducibility of quality control (QC) sample analysis. (a) Total ion current (TIC) overlay plot of QC samples. (b) CV distribution of samples in each group. The proportion of substances with CV < 0.3 in QC samples was higher than 75%, indicating excellent stability of the experimental data.

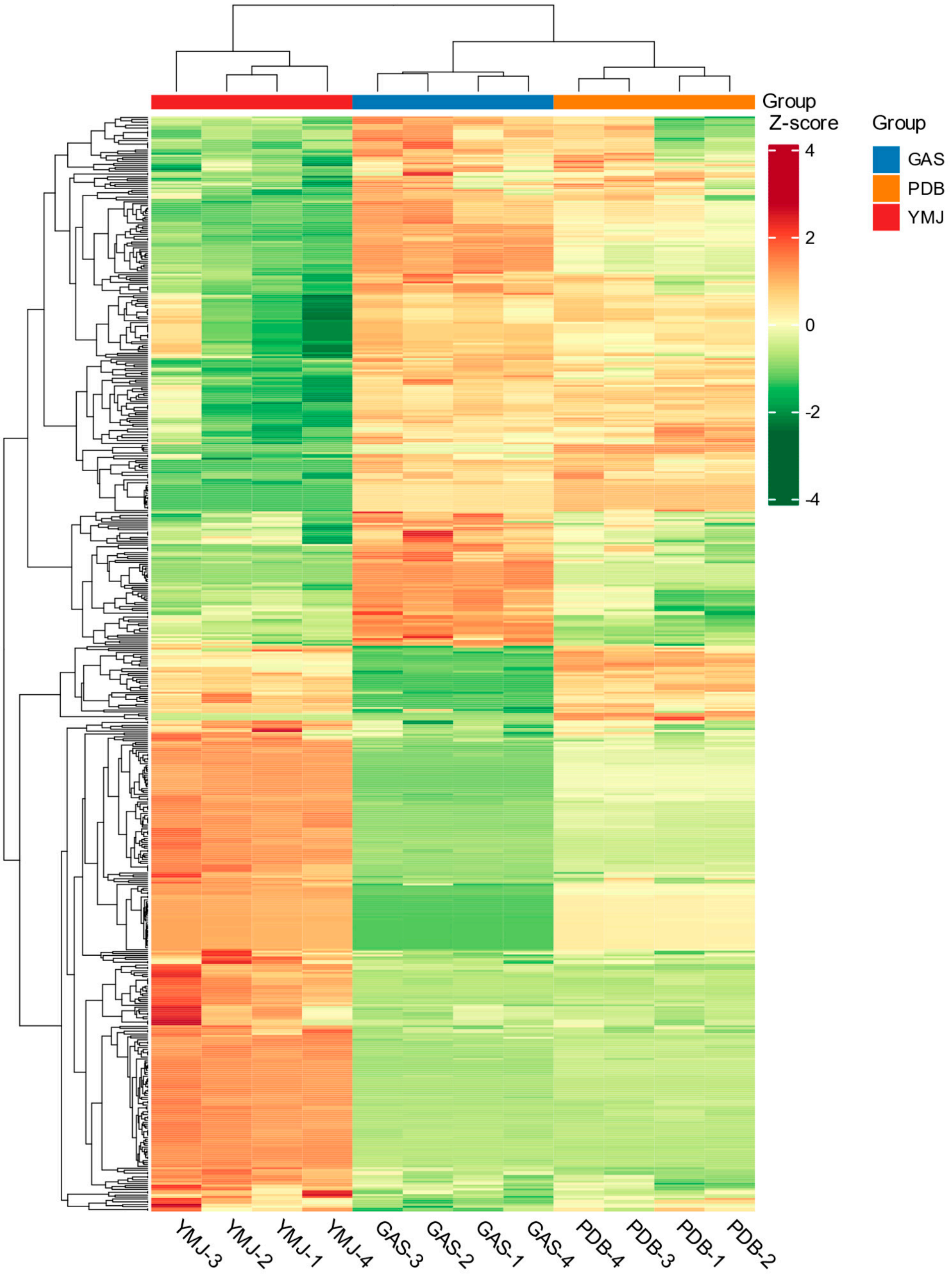

Figure S2. Hierarchical cluster analysis of DAMs across all samples.

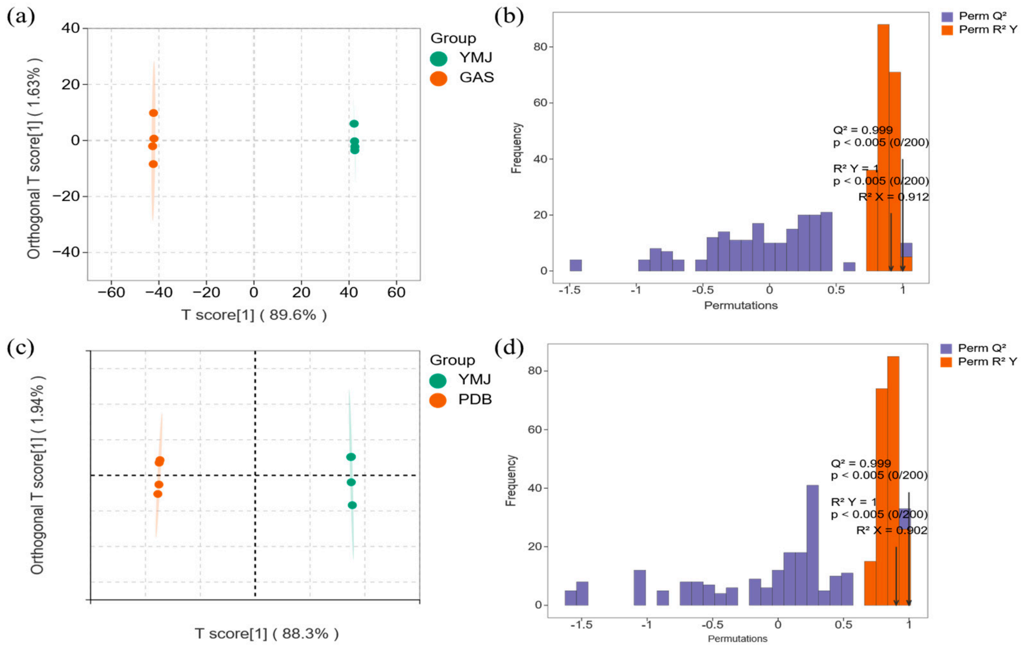

Figure S3. Validation of the OPLS-DA model. (a) Score plot and (b)validation plot for YMJ vs. GAS, Q^2=0.999, R^2Y=1, R^2X=0.912, p<0.005; (c) score plot and (d)validation plot for YMJ vs. PDB, Q^2=0.999, R^2Y=1, R^2X=0.902, p<0.005.

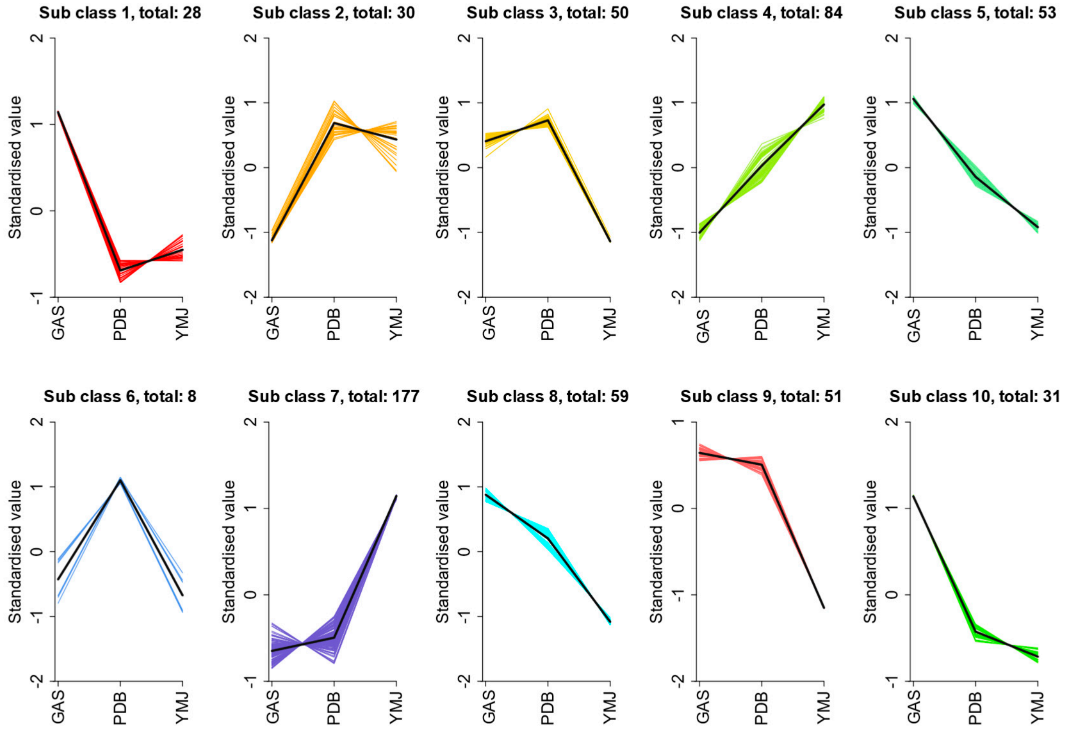

Figure S4. Trend clustering of DAMs in the three fermentation broths of *G. candidum* GC1.

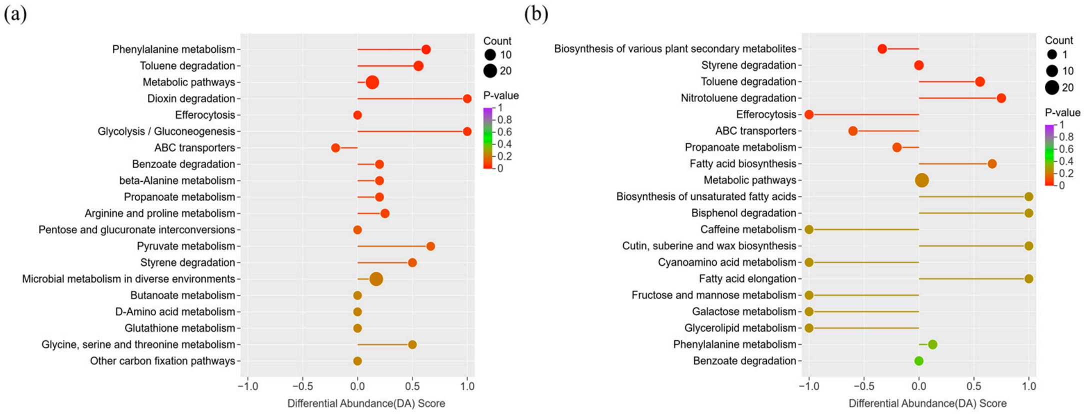

Figure S5. Differential abundance score plots for the overall analysis of metabolic pathway changes. (a) YMJ vs. PDB. (b) YMJ vs. GAS.
